# Securing Cyber–Physical Water Infrastructures: A Hybrid Intrusion Detection System for IoT Telemetry and Industrial Protocols

**DOI:** 10.3390/s26103160

**Published:** 2026-05-16

**Authors:** César López Rodríguez, Miguel Ángel Ortega Velázquez, Antonio J. Jara

**Affiliations:** Libelium Comunicaciones Distribuidas S.L., 30562 Murcia, Spain; ma.ortega@libelium.com (M.Á.O.V.); aj.jara@libelium.com (A.J.J.)

**Keywords:** industrial control systems, IoT telemetry, intrusion detection system, cybersecurity, Snort 3, critical water infrastructure, deep packet inspection, IT/OT convergence, NIS2

## Abstract

Historically, critical water infrastructures have operated with limited digitalization, relying on legacy protocols designed without intrinsic security. The rapid integration of advanced IoT telemetry into Operational Technology (OT) networks has dissolved traditional air gaps, exposing these facilities to severe cyber–physical threats. Concurrently, regulatory frameworks such as the European NIS2 Directive and the Cyber Resilience Act (CRA) now strictly mandate robust risk monitoring for essential entities. To address these challenges, this study develops a non-intrusive, hybrid Intrusion Detection System (IDS) tailored for converged IT/OT environments. Engineered upon the Snort 3 multi-threaded engine, the architecture captures both North–South and East–West traffic. A defense-in-depth rule set was constructed using threat intelligence (MITRE ATT&CK, CISA KEV) to perform Deep Packet Inspection (DPI) across legacy industrial protocols (Modbus, S7Comm, CIP) and IoT application layers (MQTT, HTTP). Experimental validation against high-volume synthetic packet captures (exceeding 170,000 packets) replicating specific manufacturer vulnerabilities (CVEs) demonstrated an improvement in the detection rate from a 0% baseline to 100%. Crucially, the system demonstrated high scalability and minimal computational overhead, processing high-volume traffic streams with zero dropped packets. This contextualized signature approach provides the deterministic security required to ensure operational continuity and regulatory compliance in modern water infrastructures.

## 1. Introduction

The management of critical infrastructures, particularly within the integrated water cycle, is undergoing a profound digital transformation driven by the deployment of the Internet of Things (IoT) and advanced sensing technologies. Historically, facilities such as Drinking Water Treatment Plants (DWTP) and Wastewater Treatment Plants (WWTP) operated with low digitalization, relying on isolated electromechanical ecosystems. Today, the demand for real-time monitoring and predictive maintenance has led to the widespread integration of distributed sensors, smart actuators, and cloud-based telemetry. This convergence between Information Technology (IT) and Operational Technology (OT) has dissolved the traditional physical isolation (air gap), transforming water management facilities into hyper-connected cyber–physical sensing environments.

While this sensor-driven modernization optimizes operational efficiency, it exposes Industrial Control Systems (ICS) to a global threat landscape. Legacy field devices and the protocols used to transmit sensor data (such as Modbus TCP, DNP3, or S7Comm) were designed prioritizing availability, inherently lacking intrinsic security mechanisms like encryption or robust authentication. Compromising the integrity of this telemetry can lead to severe physical consequences, such as chemical overdosing or uncontrolled wastewater discharges. Furthermore, new regulatory frameworks, including the European NIS2 Directive and the Cyber Resilience Act (CRA), now strictly mandate robust risk monitoring and incident reporting for essential entities, making the deployment of deterministic security measures a legal and operational imperative. Furthermore, adopting comprehensive cybersecurity governance perspectives—such as those emphasized by Catamio and Guballo [1]—highlights that ensuring system resilience and audit compliance in critical distributed infrastructures requires deterministic mechanisms capable of preventing unauthorized data manipulation and transaction errors.

Despite these regulatory mandates, a critical research gap persists regarding the operational deployment of IDS in water infrastructures. Conventional IT-centric signature models frequently lack deep protocol parsing for legacy industrial communications, leaving manufacturer-specific exploits undetected. Concurrently, probabilistic anomaly-based models tend to generate high false-positive rates, which complicate the rapid triage necessary to prevent unintended physical process shutdowns. To address these specific limitations and ensure regulatory compliance, this paper proposes the design and implementation of a non-intrusive network Intrusion Detection System (IDS) optimized for hybrid IT/OT sensing environments. The core of this proposal relies on deploying the Snort 3 multi-threaded engine with direct physical interface visibility, enabling the real-time capture of both external (North–South) and internal (East–West) traffic. Instead of relying solely on generic community signatures, the system implements a custom, defense-in-depth rule set based on active threat intelligence frameworks, such as MITRE ATT&CK and CISA’s Known Exploited Vulnerabilities (KEV) catalog. By performing Deep Packet Inspection (DPI), the proposed IDS actively searches for specific Common Vulnerabilities and Exposures (CVEs) across both legacy industrial communications and modern IoT application layers (e.g., MQTT, HTTP).

The primary objective of this article is to demonstrate how translating specific threat intelligence into highly contextualized network signatures can effectively mitigate cyber–physical attacks in the water sector. Specifically, the research aims to validate that a multi-layered inspection engine can identify and alert on exact exploitation attempts targeting both PLC control logic and IoT telemetry platforms. An additional objective is to demonstrate that this deterministic security approach can be deployed without introducing latency or disrupting the high-availability requirements of the underlying industrial processes, shifting the security coverage from a baseline of absolute vulnerability to a comprehensive detection model.

The remainder of this article is structured as follows: Section 2 presents the State of the Art, reviewing current approaches and identifying the literature gaps regarding OT and IoT sensor network security. Section 3 describes the institutional context and the real-world operational environments of the TRUEDATA project. Section 4 analyzes the specific security challenges and vulnerabilities present in converged IT/OT infrastructures. Section 5 details the architectural design of the proposed hybrid IDS and the threat intelligence methodology used for rule engineering. Section 6 presents the experimental validation, stress testing, and result analysis. Finally, Section 7 summarizes the conclusions and outlines future lines of research.

## 2. State of the Art

The paradigm shift in industrial cybersecurity is currently being driven by rapid IT/OT convergence and the inherent vulnerabilities of legacy infrastructure. Perducat et al. [2] highlighted how the integration of cloud computing and edge technologies into Operational Technology (OT) networks introduces severe scalability and security challenges. In parallel, Evripidou et al. [3] noted that cultural and organizational barriers within OT facilities often delay the adoption of modern security practices, leaving critical systems exposed. Consequently, legacy protocols encapsulated over standard TCP/IP networks remain highly vulnerable. For instance, Boeding et al. [4] demonstrated how attackers can exploit legacy exponential backoff mechanisms to trigger Denial of Service (DoS) in safety-critical environments. Recognizing these physical risks, Staves et al. [5] emphasized the need for adversary-centric testing in OT, warning that traditional IT penetration testing is often too disruptive for continuous industrial processes, thus making passive network monitoring the preferred defensive approach.

To address these threats passively, the academic focus in Network Intrusion Detection Systems (NIDS) has heavily leaned toward Artificial Intelligence (AI) and Machine Learning (ML). Yang et al. [6] provided a systematic review of anomaly-based NIDS, showing a strong research reliance on benchmark datasets. Addressing the complexities of these datasets, Fu et al. [7] and Shi et al. [8] developed advanced deep learning models capable of handling imbalanced data and multimodal network traffic. Further pushing the boundaries of AI, Qazi et al. [9] and Wang et al. [10] proposed hybrid deep-learning and TabTransformer architectures, achieving exceptionally high detection rates in simulated IT environments. Recent advancements have also expanded into IoT domains; for instance, Prakash et al. [11] illustrate the efficacy of supervised learning for smart instrumentation controllers. However, a structural comparison between AI-based and rule-based approaches reveals distinct operational trade-offs. While anomaly-based (AI) systems excel at identifying zero-day threats and complex behavioral deviations, their probabilistic, “black box” nature inherently generates false positives. In deterministic OT environments like water treatment plants—where an autonomous response to a false positive could trigger unintended physical process shutdowns—this lack of deterministic alerting complicates the rapid triage required by plant operators. Conversely, signature-based (rule) systems provide exact, actionable intelligence without false positives for known threats, though they require continuous manual updates and struggle against undocumented vulnerabilities.

Due to the strict determinism required in industrial settings, signature-based NIDS remain the operational standard. Extensive literature has compared the two leading open-source engines: Snort and Suricata. Ghazi et al. [12] and Qutqut et al. [13] provided comprehensive evaluations, historically attributing a performance advantage to Suricata in high-throughput and cloud environments due to its native multi-threading capabilities. This efficiency has driven Suricata’s adoption even in resource-constrained edge setups, as shown by Veerasingam et al. [14] in their implementation for Small and Medium Enterprises (SMEs). Nevertheless, the recent architectural overhaul of Snort has completely shifted this dynamic. As comparatively analyzed by Hoover [15] and practically implemented in virtualized Linux environments by Abdelhalim [16], Snort 3 introduces a robust multi-threaded engine, Lua-based configurations, and modular inspection. This evolution effectively closes the performance gap, making Snort 3 highly capable of performing Deep Packet Inspection (DPI) without dropping packets in complex hybrid networks.

The deployment of such deterministic inspection engines is no longer merely a technical recommendation, but a strict regulatory mandate. Ortega Velázquez et al. [17] analyzed the impact of the European Cyber Resilience Act (CRA) on the IoT lifecycle, stressing that, alongside the NIS2 Directive, there is a mandatory legal push for security-by-design and continuous risk monitoring in critical sectors. Modern connected devices and the telemetry platforms that support them must now ensure verifiable, deterministic security throughout their operational lifespans to achieve regulatory compliance.

### Comparison of the Proposed Approach with Prior Work

Current literature generally bifurcates into two distinct paradigms: probabilistic AI models and generic deterministic engines. While AI models (such as those previously discussed) provide advanced anomaly detection, they are frequently unsuited for immediate operational triage in high-availability physical processes due to their false-positive rates. Alternatively, studies evaluating deterministic engines (like Snort or Suricata) typically focus on generic IT performance metrics or rely on standard community rules. These baseline rules lack the Deep Packet Inspection (DPI) capabilities required to parse legacy industrial protocols (e.g., S7Comm, CIP) or identify manufacturer-specific vulnerabilities. This paper bridges that critical methodological gap by combining the deterministic reliability of the Snort 3 engine with a highly contextualized, defense-in-depth rule set. By actively translating specific threat intelligence (Common Vulnerabilities and Exposures—CVEs) into targeted DPI signatures for both the OT control logic and the IoT telemetry platforms, the proposed hybrid IDS directly addresses the CRA and NIS2 continuous monitoring mandates while ensuring the uninterrupted continuity of critical cyber–physical water processes.

## 3. Context of the Real Project

The research and development presented in this paper are not merely theoretical; they are integrated into the framework of a high-impact industrial project funded by public-private collaboration. This section details the institutional context of the project and the real-world operational environments that have shaped the requirements of the proposed solution.

### 3.1. The TRUEDATA Project: Secure Traceability for Hydrological Infrastructures

The system described in the following sections was developed as part of the TRUEDATA project, titled “Secure and Reliable Traceability of Data from Hydrological Infrastructures” (Trazabilidad Segura y Fiable de Datos de Infraestructuras Hidrológicas). This R&D initiative is led by the company Libelium Comunicaciones Distribuidas SL and is supported by the Spanish National Cybersecurity Institute (INCIBE) and co-funded by the European Union through the NextGenerationEU funds.

The impending enforcement of the European Cyber Resilience Act (CRA) and the NIS2 Directive imposes a paradigm shift in how industrial telemetry is managed. These regulatory frameworks strictly mandate a “security-by-design” approach and continuous vulnerability monitoring throughout the entire operational lifecycle of connected devices. However, applying these mandates to legacy water infrastructures—which often rely on unpatchable controllers and insecure protocols—presents a massive technical challenge.

**Figure 1:**
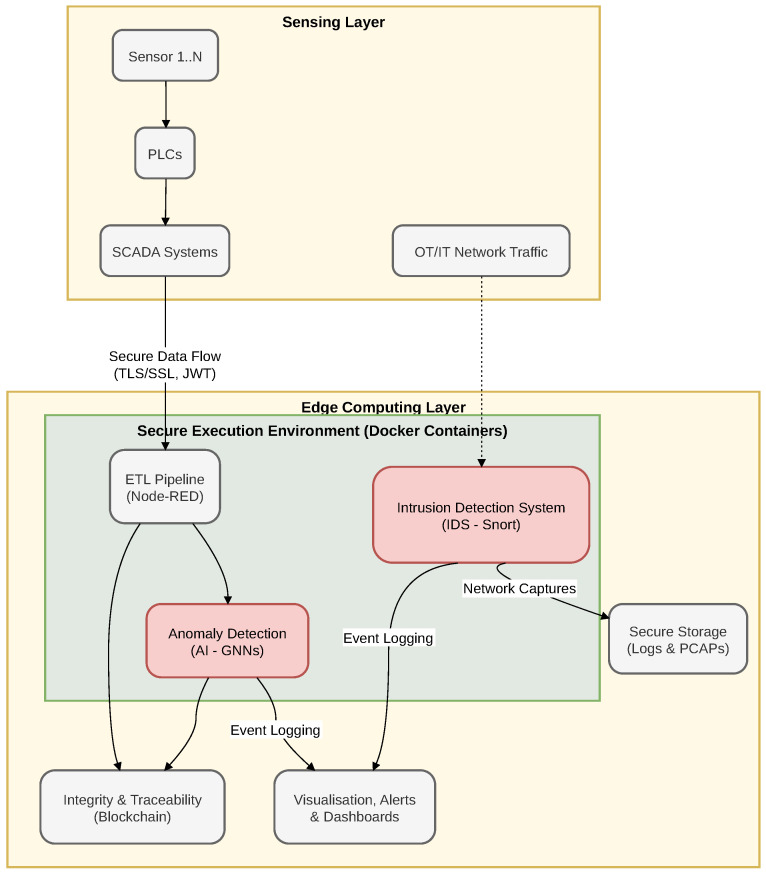
Overall architecture of the TRUEDATA project, illustrating the integration of legacy field devices, edge IDS processing, and high-level Blockchain and AI analysis modules.

The core mission of TRUEDATA is to establish a specialized ecosystem that bridges this gap, directly addressing the CRA’s requirements for continuous risk monitoring and data integrity in the water sector. The project seeks to ensure data veracity and traceability along the entire industrial data chain—from field sensors and controllers to cloud-based management platforms. To achieve this comprehensive security posture, TRUEDATA implements a synergistic technological approach at the Edge Computing Layer, as shown in Figure 1, combining three fundamental pillars:

- Blockchain Technology: Implemented to ensure the cryptographic immutability and notarization of the telemetry. This guarantees that critical events and data (such as water quality metrics) cannot be tampered with during transmission or storage, providing a reliable audit trail for regulatory compliance.
- Artificial Intelligence (AI): Utilized at higher analytical layers to model the baseline behavior of the plant and detect probabilistic anomalies. While the broader TRUEDATA architecture incorporates Graph Neural Networks (GNNs) for this purpose, the development, training, and evaluation of this AI module constitute a separate research track within the consortium and fall outside the scope of this manuscript.
- Intrusion Detection Systems (IDS): The module presented in this work, acting as the deterministic, real-time inspection engine deployed in secure Docker containers.

Within this holistic architecture, the IDS functions as the primary line of defense. To ensure scientific focus, it is crucial to delineate the specific contributions of this component from the rest of the project. The experimental validation and performance metrics presented in this paper evaluate the IDS module in strict isolation. By performing non-intrusive Deep Packet Inspection (DPI) of legacy industrial protocols, the IDS provides the continuous monitoring demanded by the CRA. It validates network integrity and identifies specific manufacturer exploits (CVEs) before the compromised telemetry data can enter the ETL (Extract, Transform, Load) pipelines or be transmitted to the higher-level AI analysis and blockchain modules.

### 3.2. Validation in Real Operational Environments

In contrast to academic prototypes confined to laboratory simulations, the detection rules and architecture proposed in this study have been tested and validated in production operational environments. The collaboration with industrial partners allowed for the deployment of the IDS in three distinct critical infrastructures, representing various facets of the integrated water cycle and Industry 4.0:

1. Drinking Water Treatment and Distribution: The solution was deployed in a Drinking Water Treatment Plant (DWTP—ETAP) and its associated pumping stations. In this scenario, the IDS monitored the communication between PLCs and the SCADA system, ensuring that process parameters (such as flow rates and chemical dosing) remained within safe operational bounds.
2. Wastewater Management: Furthermore, validation was conducted in a Wastewater Treatment Plant (WWTP—EDAR). The system performed DPI on real-time traffic to protect the instrumentation and control logic responsible for critical purification stages.
3. Private Chemical Industry: To test the versatility of the system in a highly automated environment, the deployment was extended to a chemical production plant. In this facility, water constitutes a vital raw material within an Industry 4.0 ecosystem, requiring the IDS to handle high-frequency communication between legacy industrial equipment and modern IoT platforms.

These field tests confirmed that the IDS operates as a non-intrusive probe, capable of performing deep inspection of protocols such as S7Comm, CIP, and Modbus TCP without introducing latency or affecting the availability of critical physical processes. This empirical foundation ensures that the rules described in the following sections are optimized for the high-availability requirements of actual industrial networks.

The case study focuses on the digital transformation of water treatment plants and distribution networks. In these environments, the integrity of the data is a critical requirement for both digital security and public health. The IDS architecture described in the following sections was designed to provide a non-intrusive security layer that validates the “truthfulness” of the data (hence the name TrueData) circulating between the field sensors, the PLCs, and the cloud-based management platforms.

This collaboration has allowed for the testing of the proposed rules against real traffic captures from industrial environments, ensuring that the detection patterns (described later in Section 5) are optimized to minimize false positives while maintaining the strict real-time requirements of critical process control.

## 4. Analysis of Security Challenges in Converged IT/OT Environments

Effective protection of critical infrastructures in the integral water cycle cannot rely solely on the reactive implementation of security barriers; above all, it requires comprehensive and deep knowledge of the threat landscape. A foundational premise of this study is that effective risk mitigation necessitates prior identification and comprehensive understanding. Advanced engineering projects such as TRUEDATA, which integrate Artificial Intelligence (AI), the Internet of Things (IoT), and Blockchain, have eroded traditional “security by isolation” (air-gap) barriers. This convergence exposes water treatment plants to a highly interconnected and hostile environment for which they were not originally designed.

As operational networks become increasingly distributed, identifying and managing complex threat patterns requires structured analytical approaches. For instance, recent research by Bahurmuz and Alhebi [18] demonstrates the value of clustering-based profiling to categorize complex, distributed cybersecurity environments into actionable risk models. Applying a similar philosophy of structured categorization, it is imperative to dissect the spectrum of weaknesses affecting these environments before proposing defensive strategies.

Given the extensive nature of this expanded attack surface, this study prioritizes vulnerabilities based on their quantitative severity and potential operational impact. Specifically, following standardized industry frameworks, attention is focused on High and Critical vulnerabilities—defined as having a Common Vulnerability Scoring System (CVSS) score of 7.0 or greater [19]. These targeted flaws primarily enable Remote Code Execution (RCE), logic manipulation, and Denial of Service (DoS), as they pose the most immediate threat to physical processes. Lower-impact flaws, such as simple information exposure, are cataloged but assigned secondary priority. This prioritized threat landscape directly dictates the detection capabilities of the proposed IDS, mapping the identified generic, manufacturer-specific, and platform-level vulnerabilities to the three specific defense-in-depth inspection layers detailed in Section 5.

### 4.1. Generic Vulnerabilities in ICS/OT Systems

Generic vulnerabilities constitute the basis of systemic risk in critical infrastructures. Unlike transient software bugs that can be easily patched, these weaknesses are often architectural or inherent to communication standards widely used in the industry. They persist for decades due to the extremely long life cycles of OT assets and the operational difficulty of scheduling downtime for updates. A comprehensive taxonomy of these generic vulnerabilities, mapped by their Common Weakness Enumeration (CWE) identifiers, is detailed in Table A1.

Obsolescence and Insecure Protocols: The communication backbone in Drinking Water Treatment Plants (DWTP) continues to rely on protocols designed in an era of implicit trust. Protocols such as Modbus TCP, DNP3, and EtherNet/IP fundamentally lack native encryption (CWE-311), allowing adversaries to seamlessly perform Man-in-the-Middle (MitM) attacks. In a water facility, this translates into the interception and modification of critical setpoint values—such as chlorine dosing algorithms—or the alteration of turbidity readings, effectively blinding operators to actual contamination events [20]. Furthermore, the lack of robust integrity verification facilitates Replay Attacks, where an adversary captures and retransmits valid administrative commands to execute unauthorized actions [21].

Deficient Identity Management (IAM) and Access Control: The prevalence of hardcoded credentials (CWE-798) remains a critical vulnerability; essential devices such as Programmable Logic Controllers (PLCs) and IoT gateways are frequently deployed with default passwords published in user manuals. The 2023 Aliquippa incident, where threat actors compromised Unitronics PLCs at a water authority using the default password “1111”, exemplifies the severity of this risk [22,23]. Likewise, the general lack of authentication for critical engineering functions (CWE-306) allows unauthorized actors to upload new control logic without robust validation. Additionally, poor access control mechanisms (CWE-284) often enable lateral privilege escalation once an attacker gains an initial foothold.

Memory Corruption and Input Validation: Legacy OT devices frequently suffer from improper input validation (CWE-20) and memory management flaws, such as Out-of-bounds Read (CWE-125) or Write (CWE-787). In resource-constrained field devices, sending malformed packets can easily trigger a memory corruption event, leading to Remote Code Execution (RCE) or a system-wide Denial of Service (DoS).

Web Interfaces and System Exposure. As OT devices incorporate management web servers, they inherit traditional IT flaws. The lack of sanitization leads to OS Command Injections (CWE-78) and Cross-Site Request Forgery (CWE-352), allowing attackers to force authenticated operators into executing unwanted administrative actions. Furthermore, vulnerabilities like Server-Side Request Forgery (SSRF, CWE-918) and the deserialization of untrusted data (CWE-502) allow external threat actors to bypass perimeter firewalls and reconstruct malicious objects inside the control network. This is exacerbated by Information Exposure flaws (CWE-200), where error banners leak internal IPs and software versions, aiding the attacker’s reconnaissance phase.

Network Architecture and Emerging Threats: The increasing exposure of operational services to the Internet acts as a primary vector for modern attacks. Search engines like Shodan routinely reveal thousands of publicly accessible water systems [24]. The lack of internal segmentation facilitates frictionless lateral movement from compromised corporate networks. Finally, the sector faces the alarming rise of OT-targeted Ransomware, which specifically seeks to encrypt SCADA servers, alongside sophisticated supply chain attacks that embed malware into legitimate vendor updates [25,26].

### 4.2. Manufacturer-Specific Vulnerabilities (OT Hardware)

The water sector is characterized by marked technological heterogeneity. While large treatment plants tend to standardize their infrastructure using equipment from the “Big Four”, highly dispersed distribution networks introduce a vast variety of specialized, niche devices. This analysis reveals that as modern hardware platforms evolve to offer better functionality, they paradoxically introduce new, exploitable attack surfaces. A detailed compilation of critical Common Vulnerabilities and Exposures (CVEs) affecting specific manufacturers is provided in Table A2.

Major Automation Manufacturers: Within the Siemens ecosystem, vulnerabilities in telemetry components like the TeleControl Server Basic have allowed SQL injections that compromise the central distribution database, while local privilege escalation flaws affect the S7-1500 controllers. Schneider Electric presents ongoing risks in its Modicon PLCs, particularly concerning buffer overflows in Modbus packet parsing and unauthorized logic upload capabilities (Write Strategy) via the UMAS protocol. Moreover, the EcoStruxure platform has been plagued by insecure data deserialization and SSRF vulnerabilities [27]. Meanwhile, Rockwell Automation maintains severe risks associated with legacy debugging features (WDB agents) enabled by default, which expose memory functions and allow RCE via buffer overflows in the CIP protocol [28]. Furthermore, ABB’s AC500 PLCs have suffered from directory traversal vulnerabilities that allow authenticated users to inject commands as root [29].

Specialized and Niche Manufacturers: Equipment from smaller or specialized manufacturers often exhibits severe oversights. Web servers built into WAGO or Beckhoff controllers have suffered from buffer overflows requiring zero authentication to exploit. Similarly, Omron and Phoenix Contact controllers have presented out-of-bounds reads and arbitrary file replacement vulnerabilities. SCADA and HMI platforms are equally vulnerable: Inductive Automation’s Ignition Gateway has been exposed to the import of malicious Python 3 scripts with SYSTEM privileges, AVEVA’s Edge platform suffers from weak cryptography, and PTC’s KEPServerEX is prone to resource exhaustion (DoS) when flooded with tag requests.

Smart Metering and Operator Platforms: In the realm of Advanced Metering Infrastructure (AMI), providers like Xylem (Sensus) and Itron face risks such as the disclosure of default encryption keys in wireless M-Bus networks and critical SQL injections in third-party IoT platforms, severely endangering billing data integrity [30,31]. Finally, operator-centric platforms like Suez’s Aquadvanced inherit classic XML External Entity (XXE) injections from their integrated components, allowing adversaries to read local server files [32].

### 4.3. Platform and Modern Software Vulnerabilities

Advanced digitalization initiatives heavily rely on a Cloud-Native and Edge computing technology stack. This includes the extensive use of containers, orchestration frameworks, and time-series databases. While these technologies provide immense analytical power, they introduce a distinct set of IT-centric risks into the OT realm. The complete list of identified vulnerabilities affecting this modern software stack is detailed in Table A3.

Orchestration and Containers: The adoption of Docker and Kubernetes facilitates the agile deployment of microservices but introduces the severe risk of container escapes. Vulnerabilities in container runtimes (such as runc) can allow an attacker to break the isolation boundaries. Furthermore, Docker has presented flaws leaking sensitive secrets in its logs, while insecure configurations in Kubernetes (e.g., Image Builder) can enable default root credentials on cluster nodes. Additionally, SSRF flaws in the kube-controller-manager can expose internal network access to external entities [33,34]. Edge cloud environments like AWS IoT Greengrass and Azure IoT Edge have similarly reported critical RCE flaws.

Visualization and Databases: Ubiquitous data components like Grafana and InfluxDB are fundamental for plant operators but have revealed critical flaws. Grafana has suffered from vulnerabilities allowing full identity spoofing (via SCIM) and RCE through SQL injections in underlying components like DuckDB. Such flaws allow attackers to manipulate dashboards or conceal alarms during an ongoing cyber–physical attack [35]. Similarly, databases like InfluxDB have exposed administrator API tokens in clear text [36], while TimescaleDB has faced privilege escalation via search_path manipulation. In-memory stores like Redis have also reported RCE vulnerabilities due to buffer overflows in their Lua libraries.

IoT Connectivity and Messaging: The integration of sensor data often relies on lightweight tools like Node-RED and MQTT brokers. Node-RED, if left insecure by default, has presented critical RCE vulnerabilities due to a lack of authentication, as well as directory traversal flaws allowing the reading of arbitrary system files [37]. In parallel, messaging hubs are highly susceptible to DoS and manipulation. Mosquitto brokers have suffered from memory leaks via repetitive PUBLISH packets with QoS 2 and out-of-bounds memory access through malicious SUBACK packets [38]. Other enterprise brokers like HiveMQ have been crashed using malicious JWE tokens, while RabbitMQ faced critical RCE flaws due to insecure deserialization in the OpenWire protocol.

## 5. Architectural Design of the Hybrid IDS

To effectively counter the cyber–physical threats identified in the previous analysis, a robust, deterministic, and highly specialized defense mechanism is required. This section details the overall architectural design of the proposed hybrid Intrusion Detection System (IDS) and the threat intelligence methodology used to develop its custom ruleset.

**Figure 2:**
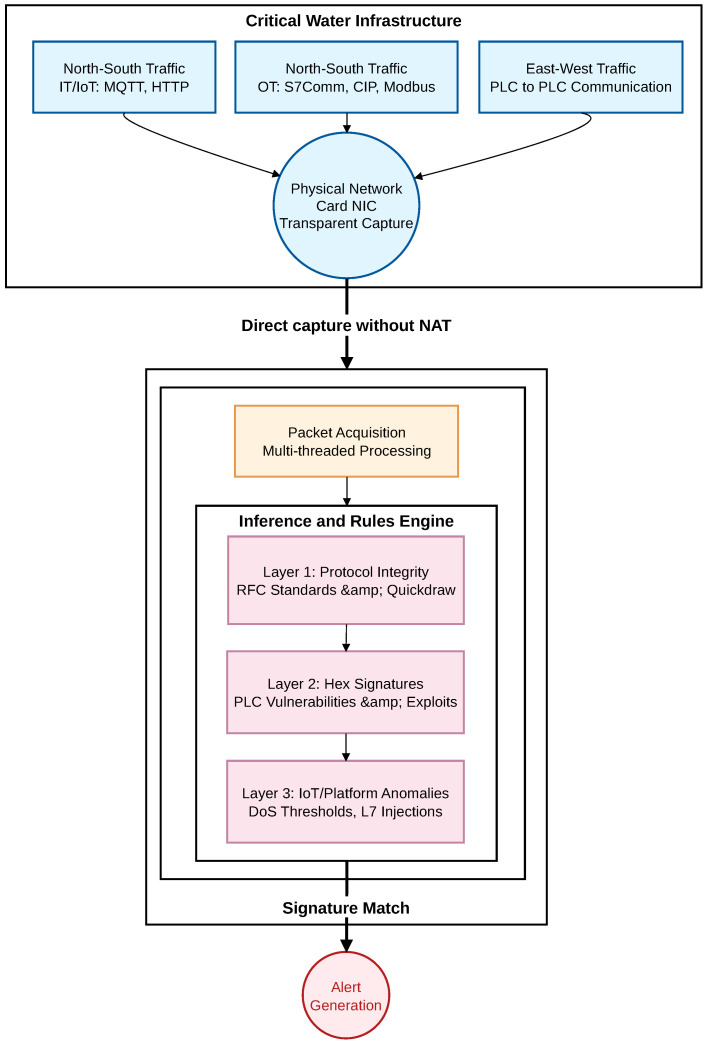
Overall architectural design of the proposed hybrid IDS, detailing the network interfaces and the inspection flow (Source: Own elaboration).

As illustrated in Figure 2, the system is strategically deployed to maintain full visibility over both the IT and OT network segments, ensuring that the critical telemetry data is inspected before reaching the higher-level analysis platforms. Specifically, the architecture is situated at the edge computing layer, acting as a secure checkpoint between the physical shop floor (sensors, PLCs, and SCADA systems) and the upper-level management services. Raw network traffic, encompassing both legacy operational protocols and modern IoT application data, is passively mirrored to the containerized Snort 3 engine. Within this secure execution environment, the traffic undergoes real-time Deep Packet Inspection (DPI). Verified, benign telemetry is permitted to continue its flow toward the ETL pipelines for subsequent AI and Blockchain processing. Conversely, any malicious payload or protocol violation instantly generates alerts and network packet captures (PCAPs), which are securely stored to facilitate immediate operator triage.

### 5.1. Core Engine: Snort 3 and Deterministic Inspection

At the core of the proposed architecture lies Snort, a widely adopted, open-source Network Intrusion Detection and Prevention System (NIDS/NIPS) originally developed by Sourcefire and currently maintained by Cisco. Traditionally, Snort operates by performing real-time traffic analysis and packet logging, using a signature-based rule engine to detect malicious activity by comparing network packets against a database of known threat profiles.

While highly effective in conventional IT environments, deploying an IDS in an Operational Technology (OT) network—such as a water treatment plant—requires strict adherence to the high-availability and zero-latency constraints outlined in guidelines like NIST SP 800-82 Rev. 3 [39]. To meet these stringent requirements, this project leverages the completely re-engineered Snort 3 architecture [40].

Unlike its predecessors, Snort 3 introduces several critical architectural improvements for industrial environments:

- Native Multi-threading: It allows the engine to process multiple packet pipelines simultaneously across different CPU cores. This ensures that the deep inspection of high-volume IoT telemetry (e.g., thousands of MQTT messages per second) does not bottleneck the network interface, thereby avoiding any latency in the critical physical process control.
- Advanced Protocol Parsers: Snort 3 includes modernized inspectors capable of normalizing and dissecting complex, encapsulated IT/OT protocols before rule evaluation, reducing evasion techniques used by attackers.
- LuaJIT Configuration: The transition to a Lua-based configuration provides dynamic scripting capabilities, allowing the IDS to load specific rulesets dynamically and optimize memory allocation when deployed in resource-constrained edge computing environments.

By deploying Snort 3 in a passive, out-of-band monitoring mode (via port mirroring or network TAPs), the system acts as a non-intrusive deterministic probe. It guarantees continuous Deep Packet Inspection (DPI) without altering the state or timing of the underlying industrial communications.

### 5.2. Threat Intelligence Integration and Rule Engineering Methodology

The effectiveness of a deterministic IDS fundamentally depends on the quality, context, and precision of its underlying ruleset. Relying solely on generic community rules typically yields high false-positive rates and fails to detect zero-day or manufacturer-specific industrial exploits. Therefore, this study proposes a rigorous, intelligence-driven methodology for engineering the custom TRUEDATA ruleset.

The rule engineering pipeline integrates several authoritative threat intelligence frameworks to systematically translate abstract vulnerabilities into actionable network signatures:

1. Vulnerability Identification and Classification: The process begins by continuously monitoring the National Vulnerability Database (NVD) [41] and the CVE Program [42] for newly disclosed flaws affecting specific OT and IoT assets. Each CVE is analyzed to understand its root cause using the Common Weakness Enumeration (CWE) framework [43], identifying if the flaw is due to an out-of-bounds read, hardcoded credentials, or improper input validation.
2. Exploitability and Prioritization: Given the vast number of published CVEs, rule development is prioritized using the Known Exploited Vulnerabilities (KEV) Catalog maintained by CISA [44]. Vulnerabilities that have been actively weaponized by threat actors in the wild receive immediate attention for signature creation. Specifically, the selection criteria for the rule set prioritized CVEs that met the previously established High or Critical severity threshold (CVSS ≥ 7.0), affected widely deployed infrastructure (e.g., Siemens, Rockwell, Schneider), and presented viable attack vectors from the network layer without requiring prior physical access.
3. Behavioral Modeling: Once a critical vulnerability is selected, the attacker’s expected network behavior is mapped using the **MITRE ATT&CK for ICS** framework [45]. This ensures that the resulting Snort rule identifies specific malicious payloads while simultaneously correlating with specific tactical objectives.
4. Signature Engineering: Finally, the intelligence is translated into a highly specific Snort 3 rule. The rule header defines the exact protocol, source/destination ports, and traffic direction, while the rule body uses advanced payload modifiers (content, pcre, byte_test) to deterministically match the byte-level footprint of the exploit.

By following this systematic pipeline, the proposed IDS shifts from a generic anomaly detector to a highly contextualized defense mechanism, capable of instantly triaging and alerting plant operators to precise exploitation attempts.

### 5.3. Defense in Depth Strategy: The Three Rule Layers

The application of the aforementioned threat intelligence methodology culminates in a structured hierarchical model. The generation of Snort alerts within this system is not conceived as an arbitrary accumulation of signatures, but rather as a direct technical response to the specific vulnerability taxonomy identified in Section 4 (spanning from generic IT/OT flaws to specific hardware and cloud-native risks).

To ensure the operational reliability of the IDS and minimize alert fatigue, this custom ruleset is organized following a strict Defense-in-Depth paradigm. Instead of evaluating all network traffic against a flat, unorganized list of rules, the inspection logic is segmented to progressively filter basic anomalies, structural protocol violations, and ultimately, complex payload exploits.

**Figure 3:**
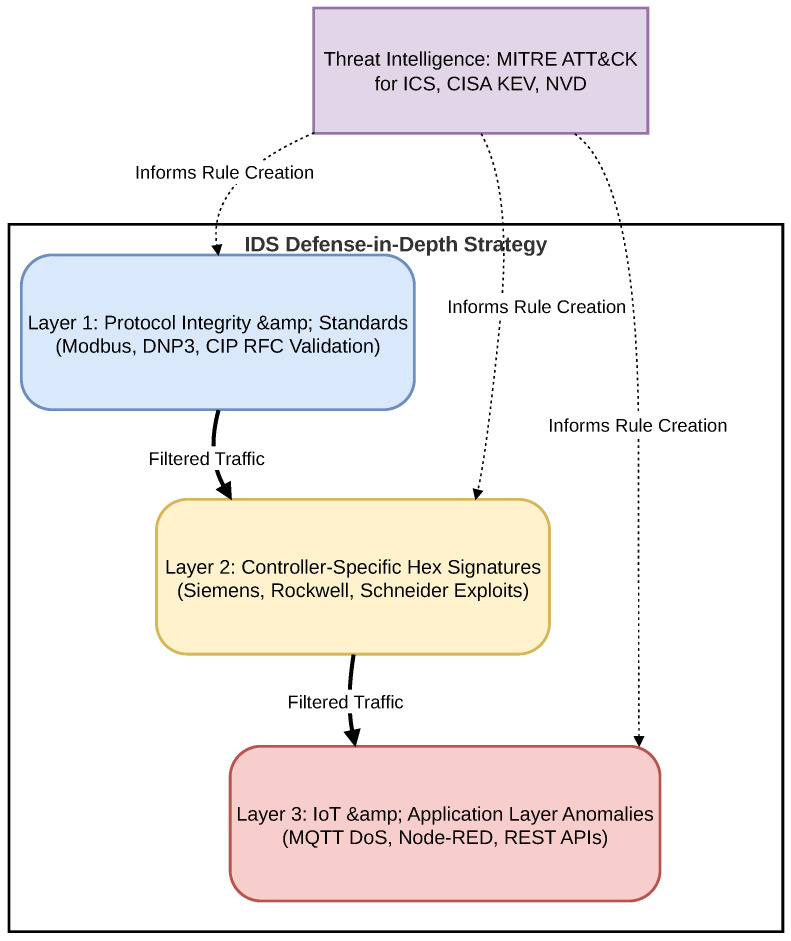
Defense-in-depth rule engineering strategy, driven by Threat Intelligence sources to cover multiple layers of the OT/IoT architecture.

As illustrated in Figure 3, this programming strategy is divided into three distinct depth layers, which are detailed below:

#### 5.3.1. Layer 1: Protocol Integrity and Standards (Quickdraw)

This layer acts as the first line of defense against generic vulnerabilities. Since protocols like Modbus TCP or DNP3 lack intrinsic security, it is vital to ensure that traffic strictly adheres to RFC specifications to avoid buffer overflows (classified under CWE-125 and CWE-787 [43]) or malformed command injections.

To achieve this, the Digital Bond Quickdraw rule repository [46], an industry standard, has been integrated and adapted. The underlying logic is common: validating header lengths and function codes. Any packet that deviates from the expected structure is preemptively treated as an exploitation attempt, regardless of its payload.

#### 5.3.2. Layer 2: Hexadecimal Signatures for Specific Controllers

To mitigate the manufacturer vulnerabilities described in Section 3, generic rules are insufficient. An extensive library of custom rules has been developed that inspects the payload through exact hexadecimal matches, designed to identify malicious commands and specific exploits in PLCs.

**Figure 4:**
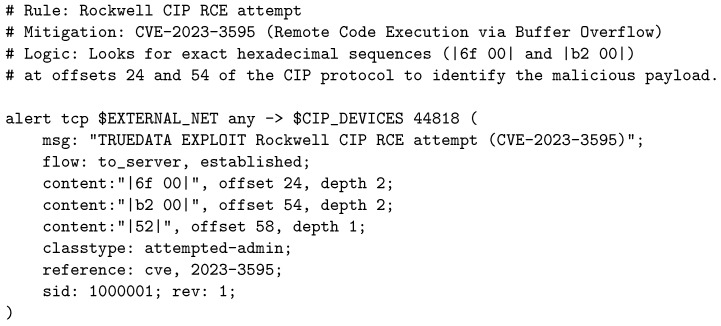
Example of a rule for detecting an RCE exploit (CVE-2023-3595) in Rockwell Automation PLCs.

**Figure 5:**
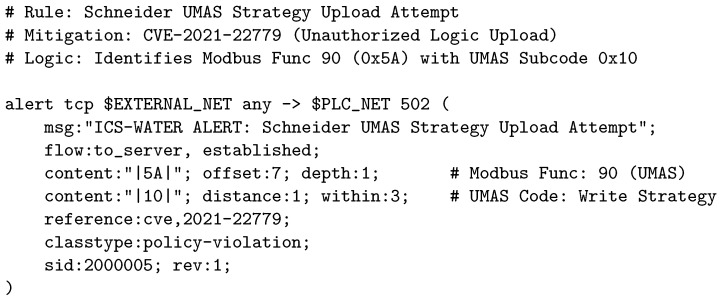
Example of a rule for detecting reprogramming in Schneider PLCs.

As a representative example of this layer, Figure 4 and Figure 5 present two of the critical rules designed to protect the most common programmable logic controllers in the water sector:

- Rockwell Automation (Figure 4): This rule mitigates the critical vulnerability CVE-2023-3595, which allows an attacker to achieve Remote Code Execution (RCE) by triggering a buffer overflow. The signature looks for specific byte sequences (|6f 00|, |b2 00|) at predetermined offsets of the CIP packet, blocking the exploit before the controller’s processor crashes.
- Schneider Electric UMAS (Figure 5): Illustrates how unauthorized reprogramming attempts are detected by inspecting the proprietary subcodes of the UMAS protocol (CVE-2021-22779), mitigating attack vectors that allow control logic manipulation.

#### 5.3.3. Layer 3: Anomalies in the IoT Application Layer and Platforms

Finally, the IT/OT convergence analyzed in the introduction introduces risks in the software layer (MQTT, REST APIs, Containers). To cover these platform vulnerabilities, rules utilizing threshold filters, regular expressions, and bit analysis have been designed, based on the official security advisories of each technology.

Signatures have been implemented to detect Path Traversal attempts in Node-RED [47], SSRF injections in ThingsBoard [48], and authentication bypass in OPC UA servers [49].

**Figure 6:**
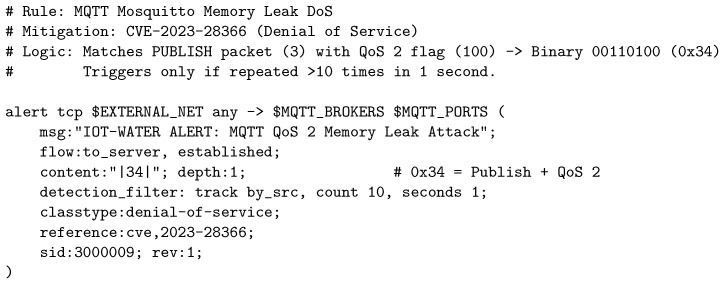
Example of a rule for DoS mitigation in IoT infrastructure.

Figure 6 shows a paradigmatic example designed to mitigate CVE-2023-28366 in Mosquitto brokers [50]. This rule demonstrates how the combination of bit-level header inspection (detecting the QoS 2 flag) with frequency filters (*detection_filter*) allows identifying complex Denial of Service (DoS) attacks that would go unnoticed by a conventional firewall.

## 6. Experimental Validation and Result Analysis

The validation of the Intrusion Detection System (IDS) in critical environments cannot rely on chance or theoretical assumptions. Therefore, a rigorous experimental validation phase was conducted to quantify the security improvements achieved by the proposed architecture.

### 6.1. Methodology and Safe Traffic Generation

To strictly avoid putting the physical processes and operational continuity of the real water treatment plants at risk, testing was not conducted by detonating live malware within the production network. Instead, a safe and highly realistic attack simulation environment was developed.

Using Python and the Scapy library, custom scripts were engineered to synthesize “bespoke” network traffic, forging packets byte-by-byte to replicate the exact digital signatures of known vulnerabilities. These synthetic attacks were compiled into controlled PCAP (Packet Capture) files.

A critical aspect of this validation was ensuring the resilience of the IDS under realistic industrial loads. The generated datasets included high-volume stress tests, with individual packet captures exceeding 156,000 and 170,000 packets. By interleaving malicious payloads within massive streams of legitimate telemetry, the tests verified that the multi-threaded Snort 3 engine could maintain deterministic, real-time inspection without dropping packets or suffering buffer exhaustion.

### 6.2. Ablation Study: Evolution of Security Coverage

**Figure 7:**
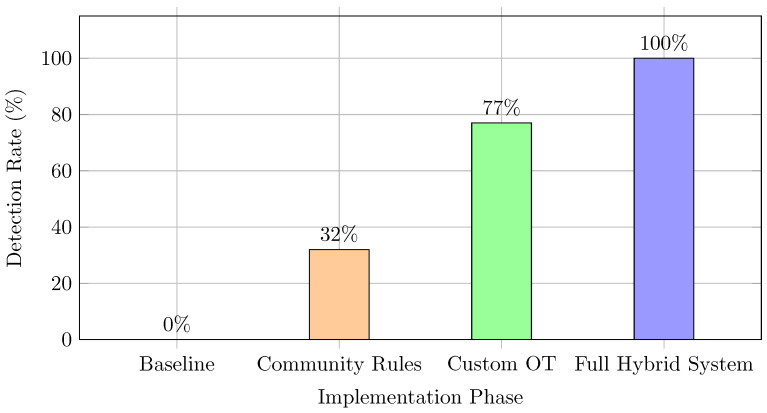
Evolution of the global security coverage and detection rate across the incremental testing phases.

The evaluation methodology was structured incrementally across four phases to isolate and measure the added value of each rule set, effectively functioning as an ablation study to evaluate the individual contribution of each architectural layer. As illustrated in Figure 7, the progression of the system’s defensive capabilities was substantial:

- Phase 1 (Baseline): In the initial state without specific detection policies, the system operated as a negative control, yielding a 0% detection rate.
- Phase 2 (Community Rules): The implementation of standard hygiene signatures (Quickdraw) raised the detection rate to 32%. While effective for generic anomalies, the system remained blind to sophisticated exploits.
- Phase 3 (Custom OT Rules): The integration of the manufacturer-specific signatures designed in this study increased the detection rate to 77%, mitigating critical vectors against field devices.
- Phase 4 (Full System): The final deployment, including the IT/IoT platform service rules, achieved a 100% detection rate against the controlled test dataset.

Furthermore, analyzing the global detection rate alone can obscure the system’s effectiveness against specific technological stacks. Therefore, a breakdown of the IDS performance across different protocol families was conducted, evaluating both legacy OT communications and modern IT/IoT layers.

**Figure 8:**
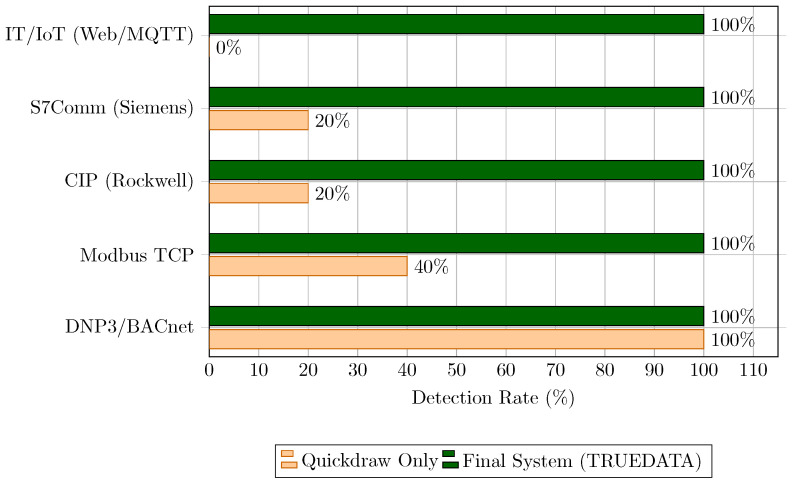
Comparison of detection efficacy by protocol family.

As depicted in Figure 8, the hybrid rule engine achieved a robust detection capability across the entire convergence spectrum. While open industrial protocols like Modbus TCP demonstrated measurable improvement from the Layer 1 structural validation, the manufacturer-specific signatures in Layer 2 were crucial for securing proprietary protocols such as Siemens S7Comm and Rockwell CIP against advanced exploits. Simultaneously, the Layer 3 inspection proved highly effective in mitigating application-layer attacks directed at IoT MQTT brokers and web-based HTTP APIs.

While the full hybrid system achieved a 100% detection rate within this controlled experimental framework, these results must be interpreted within their specific operational context. The current evaluation utilized synthetically generated traffic captures focused on proving detection efficacy. Because the dataset does not fully replicate the highly imbalanced, large-scale nature of real-world benign network traffic, the calculation of robust operational metrics—such as False Positive Rate (FPR), precision, and recall—was precluded. Future evaluations of system robustness will require advanced methodologies for handling minority class predictions and imbalanced data, such as those demonstrated by Sarmini et al. [51]. Furthermore, the current experimental setup does not evaluate advanced evasion techniques or encrypted traffic (e.g., TLS 1.3 encapsulation). Because the proposed system fundamentally relies on Deep Packet Inspection (DPI), pervasive encryption without proper key brokering or traffic decryption strategies would obscure payload visibility, necessitating complementary architectural adaptations.

## 7. Conclusions

The digital transformation of the integrated water cycle has demonstrated that IT/OT technological convergence exposes critical infrastructures to severe vulnerabilities that traditional defense mechanisms fail to mitigate. This study empirically proves that generic security solutions are insufficient for protecting these hybrid sensing and control environments. The experimental evaluation revealed that an Intrusion Detection System (IDS) relying solely on standard community rules achieved a detection rate of only 32%, remaining blind to specific exploits targeting PLC control logic and IoT platforms. In contrast, the implementation of the proposed Snort 3-based architecture, equipped with a defense-in-depth strategy and contextualized signatures (TRUEDATA), achieved a 100% effectiveness rate in detecting the evaluated attack vectors.

Furthermore, it is concluded that effective security in the water sector requires unified protection encompassing both legacy industrial protocols (e.g., Modbus, S7Comm, CIP) and the platform services layer. Security risks can originate in IT components (such as IoT telemetry platforms or containers) and propagate into the operational network. The direct traceability achieved between identified risks (CVE vulnerabilities) and network rules allows operators to drastically reduce incident triage and response times. Additionally, the containerized deployment using the host network mode has proven to be a robust and non-intrusive solution, guaranteeing physical traffic visibility without compromising the availability of critical processes.

Despite the demonstrated efficacy of this architecture, this study acknowledges several operational limitations. The empirical validation was conducted under controlled experimental conditions; transitioning this architecture to large-scale, highly distributed water infrastructures introduces practical deployment challenges. Specifically, maintaining a deterministic IDS requires continuous manual updates to the rule set to adapt to rapidly evolving threats, which generates an ongoing administrative overhead. Furthermore, while the technical defense layers are robust, they must be contextualized within broader socio-technical frameworks. Human-centric vulnerabilities and behavioral factors—such as those analyzed by Aljohani and Alnahdi [52] regarding technology dependencies and cognitive overload—play a critical role in system resilience. An over-reliance on deterministic alerts without adequate operator training and situational awareness can lead to alert fatigue, ultimately undermining the cyber–physical security posture.

To address these limitations and extend the capabilities of this research, several avenues for future work have been identified. First, the evolution of the system towards an Intrusion Prevention System (IPS) capable of actively blocking malicious traffic is proposed; this transition will require an exhaustive study on the impact of latency and false positives to prevent unintended physical disruptions. Second, feeding the deterministic IDS alerts into Artificial Intelligence (Machine Learning) models is suggested to establish traffic baselines and detect unknown probabilistic anomalies. Finally, the integration of this telemetry into Security Information and Event Management (SIEM) platforms will enable advanced incident correlation, providing a holistic view of security across the entire cyber–physical infrastructure. 

## Data Availability

The original contributions presented in the study are included in the article, further inquiries can be directed to the corresponding author.

## Abbreviations

The following abbreviations are used in this manuscript:

IoT	Internet of Things
OT	Operational Technology
IDS	Intrusion Detection System
DPI	Deep Packet Inspection
CVE	Common Vulnerabilities and Exposures
IT	Information Technology
DWTP	Drinking Water Treatment Plant
WWTP	Wastewater Treatment Plant
ICS	Industrial Control Systems
TCP/IP	Transmission Control Protocol/Internet Protocol
HTTP	Hypertext Transfer Protocol
MQTT	Message Queuing Telemetry Transport
REST	Representational State Transfer
API	Application Programming Interface
DoS	Denial of Service
CPPS	Cyber–Physical Production Systems
IIoT	Industrial Internet of Things
NIDS	Network Intrusion Detection Systems
SME	Small and Medium-sized Enterprises
R&D	Research and Development
INCIBE	Spanish National Cybersecurity Institute
SOC	Security Operations Center
AI	Artificial Intelligence
ETAP	Drinking Water Treatment Plant
PLC	Programmable Logic Controller
SCADA	Supervisory Control and Data Acquisition
EDAR	Wastewater Treatment Plant
IAM	Identity and Access Management
CWE	Common Weakness Enumeration
RCE	Remote Code Execution
CSRF	Cross-Site Request Forgery
SSRF	Server-Side Request Forgery
XXE	XML External Entity
NAT	Network Address Translation
NIC	Network Interface Card
LAN	Local Area Network
NVD	National Vulnerability Database
KEV	Known Exploited Vulnerabilities
PCAP	Packet Capture
IPS	Intrusion Prevention System
SIEM	Security Information and Event Management

## Appendix A. Taxonomy of Identified Vulnerabilities

**Table A1:** Complete Taxonomy of Identified Generic Vulnerabilities.

ID (CWE/Threat)	Description and Operational Impact
CWE-798 Hardcoded Credentials	Use of hardcoded passwords in firmware or software. Allows trivial administrative access if the attacker knows the default keys (e.g., user manuals).
CWE-311 Missing Encryption	Transmission of sensitive command and control data in clear text. Facilitates sniffing and manipulation of process variables via Man-in-the-Middle attacks.
CWE-306 Missing Authentication	Execution of critical configuration changes, firmware updates, or emergency stops without verifying the identity of the requester.
CWE-20 Improper Input Validation	Lack of input data sanitization in OT/IT interfaces, allowing code injections and Denial of Service (DoS) conditions.
CWE-284 Improper Access Control	Insufficient restrictions for authenticated users. Allows horizontal or vertical privilege escalation within the control network.
CWE-125 Out-of-bounds Read	Reading memory past the allocated buffer, allowing an attacker to access sensitive information residing in the device’s RAM.
CWE-787 Out-of-bounds Write	Writing memory past the buffer, which can corrupt critical operation data or allow Remote Code Execution (RCE).
CWE-200 Information Exposure	Leakage of technical information (software versions, internal IP addresses) in error messages or banners that facilitate the reconnaissance phase.
CWE-352 CSRF	Web vulnerability that allows forcing an authenticated user to perform unwanted actions on the system administration interface.
CWE-78 OS Command Injection	Insecure construction of operating system commands in web applications or SCADA, allowing arbitrary command execution on the server.
CWE-918 SSRF	Induces the server to make arbitrary HTTP requests to internal or external resources, bypassing perimeter firewalls.
CWE-502 Deserialization of Untrusted Data	Processing serialized data without prior validation, which can lead to remote code execution when reconstructing malicious objects.
CWE-400 Resource Consumption	Deliberate exhaustion of CPU, memory, or bandwidth via malicious requests, causing a Denial of Service (DoS).
Threat: OT Ransomware	Malicious encryption of engineering stations, historical databases, and SCADA servers, paralyzing operation and demanding ransom.
Threat: Supply Chain	Compromise of trusted vendors to introduce malware into legitimate software updates before they reach the end user.

**Table A2:** Identified Critical Manufacturer Vulnerabilities.

Manufacturer	Product	CVE/ID	Flaw Description
Siemens (Munich, Germany)	TeleControl Server	CVE-2025-27540	SQL Injection allowing DB read/write and potential RCE.
	S7-1500/TIA	CVE-2024-36339	Flaw in AMD rendering libraries allows local privilege escalation.
Rockwell (Milwaukee, WI, USA)	ControlLogix 1756	CVE-2025-7353	Debug agent (WDB) exposes memory functions allowing remote control.
	Arena Sim	CVE-2025-7032	Memory abuse when opening manipulated simulation files (RCE).
	PLCs (CIP)	CVE-2023-3595	Remote Code Execution via Buffer Overflow in the CIP protocol.
Schneider (Rueil-Malmaison, France)	Modicon M340	CVE-2024-8937	Buffer overflow in Modbus packet handling (RCE).
	EcoStruxure Geo	CVE-2025-54923	Insecure data deserialization allowing remote code execution.
	EcoStruxure Geo	CVE-2025-54924	SSRF allowing unauthorized access to internal server data.
	PLCs (UMAS)	CVE-2021-22779	Unauthorized logic upload (Write Strategy) via Modbus Func 90.
ABB (Zurich, Switzerland)	AC500 V3 PLC	CVE-2024-12430	Directory traversal allows authenticated users to inject commands as root.
Omron (Kyoto, Japan)	NJ/NX Series	CVE-2025-1384	Privilege violation in Sysmac Studio allowing code execution.
	Sysmac Studio	CVE-2025-0591	Out-of-bounds read in CX-Programmer file parser.
Phoenix (Blomberg, Ger.)	PLCnext/AXC	CVE-2025-41667	Replacement of critical system files with low privileges.
WAGO (Minden, Germany)	PFC100/200	CVE-2025-41730	Buffer overflow in management web server without requiring authentication.
Unitronics (Airport City, Israel)	UniStream	CVE-2024-27767	Improper authentication granting full administrative control.
Mitsubishi (Tokyo, Japan)	MELSEC iQ-F	CVE-2025-7405	Flaw in Modbus TCP packet processing allows command execution.
Yokogawa (Tokyo, Japan)	FAST/TOOLS	CVE-2025-66608	Incorrect URL validation facilitating theft of sensitive files.
	GX Recorders	CVE-2025-1863	Lack of authentication in communication functions allows data manipulation.
Emerson (St. Louis, MO, USA)	Ovation	CVE-2024-32752	Lack of authentication in critical protocols allows RCE on the controller.
Beckhoff (Verl, Germany)	TwinCAT 3	CVE-2025-41726	Arbitrary code execution via Device Manager web service.
Inductive (Folsom, CA, USA)	Ignition Gateway	CVE-2025-13911	Import of malicious Python scripts with SYSTEM privileges.
AVEVA (Cambridge, UK)	Edge/InTouch	CVE-2025-9317	Weak cryptography allowing brute-force attacks on passwords.
PTC (Boston, MA, USA)	KEPServerEX	CVE-2024-6098	Uncapped resource allocation (DoS) with large volumes of tags.
Festo (Esslingen, Germany)	CPX Terminal	CVE-2025-2024	RCE via parsing of malformed SKP configuration files.
Xylem (Washington, DC, USA)	FlexNet/RNI	XPSA-2024-015	Disclosure of the default encryption key used in the wM-Bus network.
Itron (Liberty Lake, DC, USA)	Smart Meters	CVE-2025-63624	Critical SQL Injection in third-party IoT platform integrated with meters.
Suez (Paris, France)	Aquadvanced	CVE-2021-46660	XXE vulnerability allowing reading of local server files.

**Table A3:** Vulnerabilities in the Software Stack and Platforms.

Component	Category	CVE/ID	Risk Description
Grafana	Visualization	CVE-2025-41115	Critical Vulnerability (10.0). Full identity spoofing via SCIM.
	Visualization	CVE-2024-9264	RCE via injection in SQL expressions (DuckDB component).
InfluxDB	Database	CVE-2024-30896	Recovery of administrator tokens in clear text via API.
Kubernetes	Orchestration	CVE-2024-9486	Image Builder enables default root credentials on cluster nodes.
	Orchestration	CVE-2025-13281	SSRF in kube-controller-manager allows access to internal network.
Docker	Containers	CVE-2025-3911	Reading sensitive secrets in Docker Desktop logs.
Node-RED	IoT Low-Code	CVE-2025-41656	RCE (Score 10.0) due to lack of default authentication.
	Dashboard	CVE-2021-3223	Directory traversal allowing reading of arbitrary system files.
Mosquitto	MQTT Broker	CVE-2024-10525	Out-of-bounds memory access (DoS) via malicious SUBACK packet.
	MQTT Broker	CVE-2023-28366	Memory leak causing DoS via repetitive PUBLISH packets with QoS 2.
HiveMQ	MQTT Broker	CVE-2024-29370	DoS via sending malicious JWE tokens.
RabbitMQ	Messaging	CVE-2023-46604	Insecure deserialization in OpenWire protocol allows RCE.
TimescaleDB	Series DB	CVE-2023-25149	Privilege escalation via search_path manipulation.
AWS IoT	Cloud/Edge	CVE-2025-31133	Container escape in AWS Greengrass (runc vulnerability).
Azure IoT	Cloud/Edge	CVE-2025-60711	Flaw in Azure IoT Edge allows remote code execution.
Redis	Cache/DB	CVE-2024-31449	Buffer overflow in Lua library allows RCE.
Beckhoff	TwinCAT Pkg	CVE-2024-8934	Local command injection as SYSTEM via Package Manager.
